# The effect of estuarine system on the meiofauna and nematodes in the East Siberian Sea

**DOI:** 10.1038/s41598-021-98641-1

**Published:** 2021-09-29

**Authors:** Daria A. Portnova, Lesya A. Garlitska, Alexander A. Polukhin

**Affiliations:** Shirshov Institute of Oceanology, Russian Academy of Sciences, 36, Nahimovskiy prospekt, Moscow, Russia 117997

**Keywords:** Ecology, Biodiversity, Hydrology

## Abstract

Arctic meiofauna and nematodes were examined at 12 stations in the East Siberian Sea, covering a depth range of 13–59 m and an estuarine-shelf system from the Indigirka and Kolyma rivers to the adjacent shelf. Our data reveal the low diversity of the meiofauna at the East Siberian Sea shelf. The meiobenthos abundance was influenced by river run-off and the sea bottom landscape. The samples comprised a total of 28 families and 72 genera, and the number of genera per station ranged from 15 to 32. The Comesomatidae was the dominant family with genus *Sabatieria*. Among all factors, depth, water temperature and the total organic carbon appeared to be important variables explaining spatial variations in meiofauna and nematodes abundance. Depth and river run-off were defining in controlling the density of nematodes in the study area.

## Introduction

The East Siberian Sea (ESS) is one of the continental shelf seas with the widest and shallowest shelf in the World Ocean, which has an open boundary with the Arctic Basin in its northern part^1^. The ESS is covered with ice most of the year and this is a difficult area for researching compared with the other Arctic seas. On the ESS shelf occurs flaw leads to polynya. This is part of a recurring phenomenon known as the Great Siberian Polynya, and it may be up to 200 km wide^2^. Oceanographic studies in this area have been almost entirely of a chemical or physical nature. An important feature of the ESS shelf is the aragonite undersaturation of its waters, which is due to the inflow of organic matter from thawing permafrost with continental run-off. Acidification of the ESS shelf is occurring faster than predicted and may cause a negative effect on the local ecosystem.

Study of the meiofauna in the EES began in 1973 during the 2nd Arctic expedition of the Zoological Institute Academy of Science USSR^3^. For the first time, on this expedition they collected meiobenthos in addition to macrobenthos. Sediment samples of the grain size and organic matter content were collected at the same stations as the meiofauna samples^3^. As a result of these expeditions, they inventoried the eastern part of the ESS, and in 1986 and 1989, they also inventoried the western part of the ESS for the composition and distribution of Arctic benthos^4–7^.

Meiofauna are described a size class in the benthos which pass a sieve of 1 mm mesh size and retained on a sieve of 45 mesh size, includes a specific set of taxa, with series of specific ecological and evolutionary characteristics: such as generation time, life-history traits, response time to temporal changes^8,9^. The meiofauna community structure is commonly related to abiotic gradients in sediment composition and grain content, decomposition of organic matter, seasons, salinity, temperature fluctuations, water depth, tidal exposure^9,10^. Microtopographic unevenness in abundance and diversity in meiobenthos can be generated by patchy distribution of food sources as well as biological activities, such as bioturbation and the construction of biogenic structures by other benthic organisms^11,12^.

In the autumn of 2017 (22 August to 6 October) the cruise Ecosystems of the Siberian Arctic Seas-2017 was conducted with the objective of investigating the processes occurring in the estuarine areas of the Siberian rivers and on the shelf of the epicontinental Arctic seas, which affects the Central Arctic Basin via a system of cross-shelf and cross-slope transport^13^. The study of the ecology and sampling of Kara, Laptev and ESS benthic fauna was undertaken on board the R/V “Akademik Mstislav Keldysh” in this cruise.

The export of turbid waters from Arctic rivers and coastal regions could enhance the delivery of nutrients but could also impair photosynthesis by scattering and absorbing sunlight. The study in the Yenisei Gulf and adjacent parts of the Kara Sea demonstrates how the environmental conditions acting together affect the harpacticoid copepod and nematode community structures^14,15^. Recently, the data revealed noticeable changes in diversity and composition of meiobenthic and nematode communities along the latitudinal transect in the Laptev Sea from relatively low-diversity communities near the Lena Delta to more diverse and abundant fauna in the shelf. The vicinity of the Lena Delta plays an important role in the taxonomical structuring of nematode communities. Mixing of sediments and settling of resuspended matter from Lena River discharge is caused a vertical redistribution of the sediment layers, which impact both positive and negative effects on meiofauna^10^. Due to the low study of the ESS, we need to test the hypothesis of whether the Indigirka and Kolyma rivers discharge affects the meiofauna and the community of nematodes in the ESS. Do meiofauna and nematode community densities and composition change along a bathymetric transect? Are these changes correlated with hydrophysical and hydro-chemical information available on the water column and sediment? This study of the ESS is by far the most comprehensive collection of meiobenthic data, and in this contribution, we analyzed data on the meiofauna and nematodes assemblages at 500–km latitudinal transects across the Indigirka and Kolyma Deltas areas (1) to describe the meiobenthic distribution patterns in the western ESS; and (2) to identify environmental factors controlling the genera composition and taxonomic structure of nematodes.

## Results

### Indigirka transect

**Figure 1:**
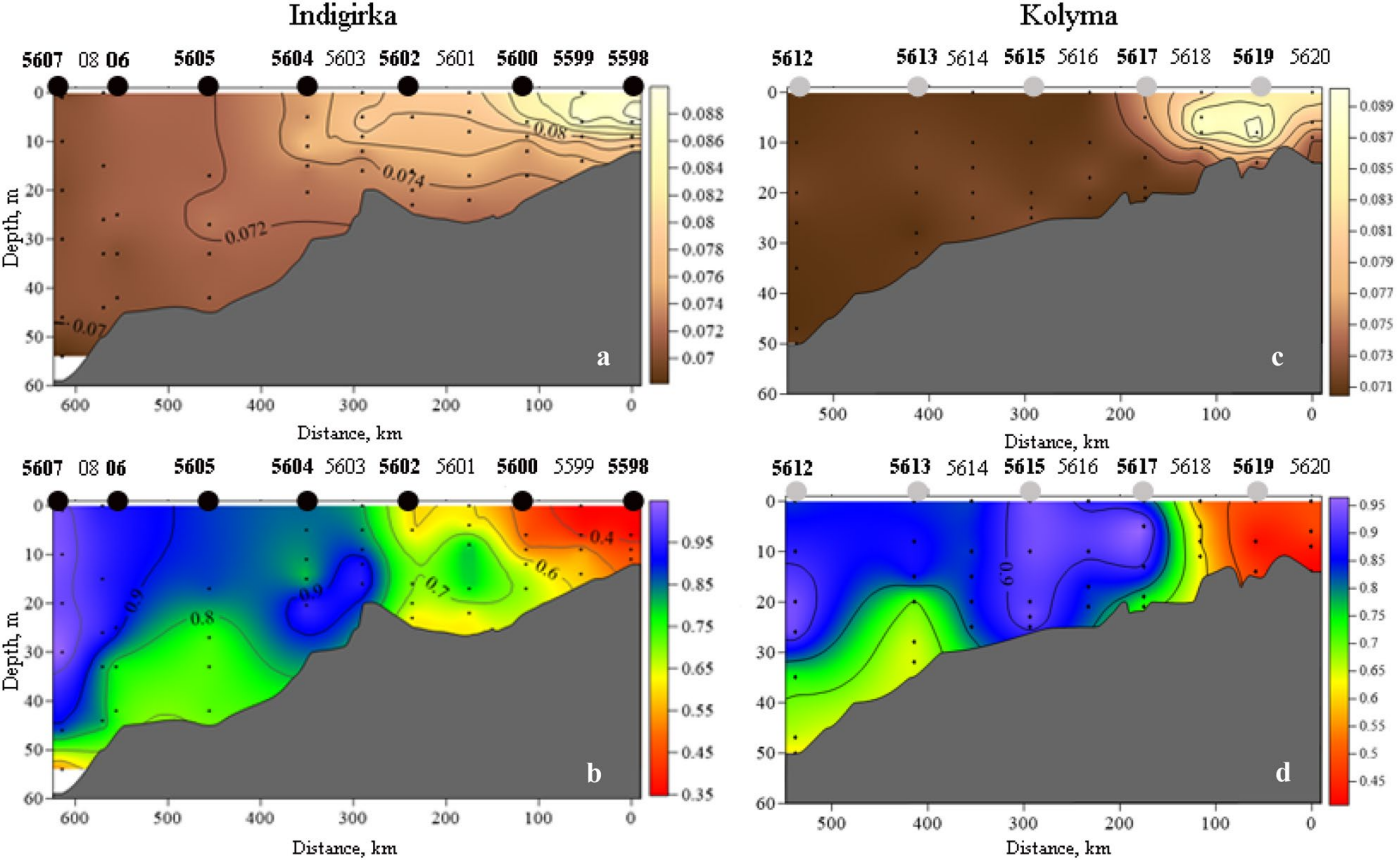
Distribution of riverine water using At/S ratio (**a**,**c**) and aragonite saturation (**b**,**d**) along the Indigirka and Kolyma transects. Maps was created with Golden Software Surfer version 8. Black circles indicate meiobenthic stations in Indigirka river, grey circles in Kolyma river.

The length of the Indigirka transect is 614 km with a depth from 14 to 58 m. It begins 12 nautical miles to the north of the Indigirka delta and ends on the continental shelf. In August 2017, the average water discharge from the Indigirka was 1355 m^3^/s, which is 3.5 km^3^ or 7% of the annual run-off. The greatest influence of the Indigirka run-off was noted at station 5598, where the At/S ratio is 0.089 (Fig. 1a).

With distance from the coast, the influence of the river run-off noticeably decreased. The frontal zone is clearly expressed up to station 5601 (200 km from the first site 5598) and has a combined type (elements of the vertical and horizontal frontal zones). An interesting feature of the water structure is the fact that the influence of the continental run-off is traced throughout the entire water column, from the surface to the bottom, which is determined by a At/S ratio of more than 0.07 units. Only in the surface and bottom layers on site 5607 is pure seawater released (the At/S ratio is 0.068). The entire water column along the section is undersaturated with aragonite, in the area of the maximum influence of the Indigirka run-off, *Ω*_*Ar*_ is 0.3–0.6, then it increases in the seaward part and reaches *Ω*_*Ar*_ = 1 at the northernmost station of the section (Fig. 1b). At stations 5605 and 5606, an upwelling effect with lower *Ω*_*Ar*_ water is noted.

### Kolyma transect

**Figure 2:**
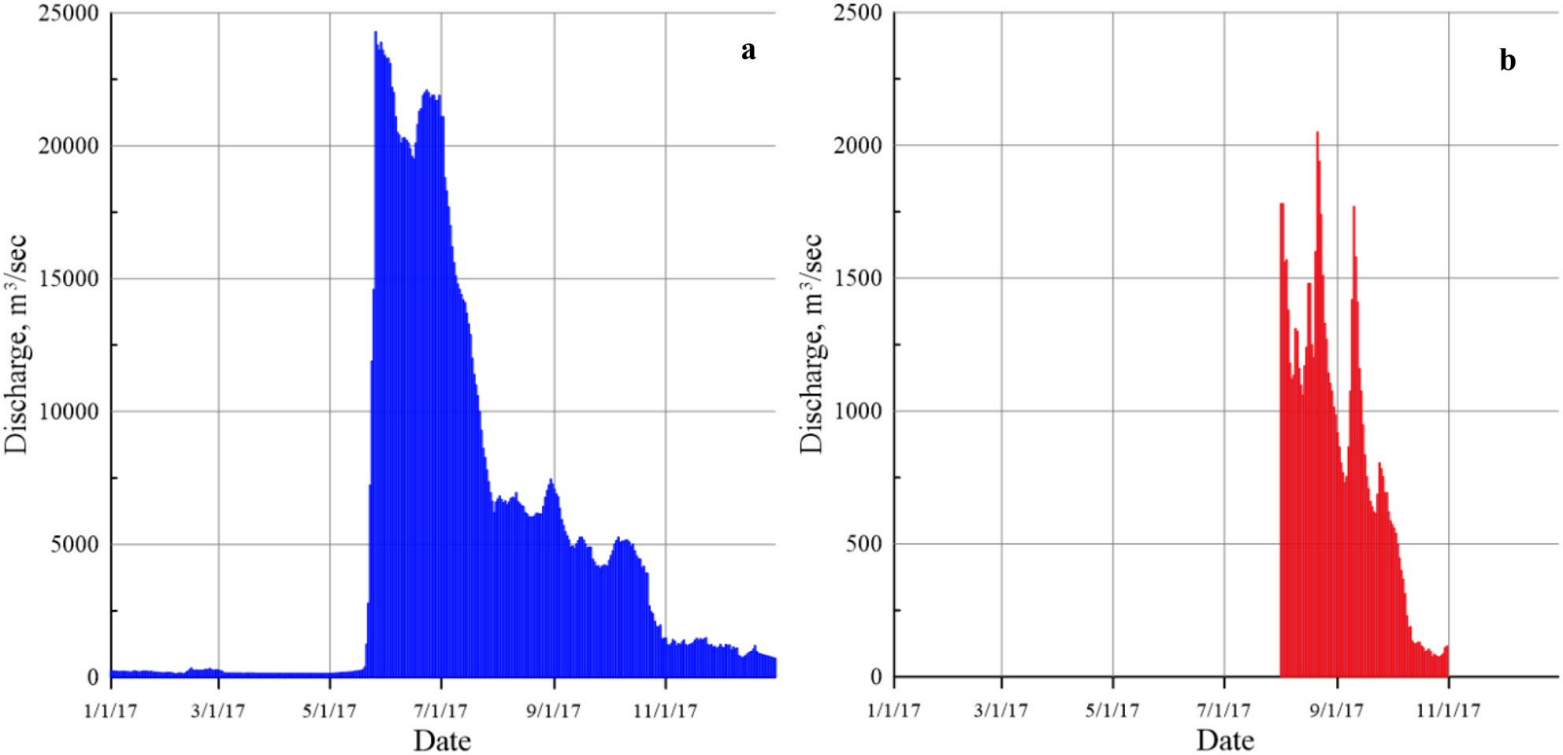
Discharge of the Kolyma (**a**) and Indigirka (**b**) Rivers in 2017. Data was obtained from the Arctic Great Rivers Observatory project web-site^62^.

The length of the Kolyma transect is 538 km, with a depth ranging from 17 to 50 m. In 2017, the average annual run-off of the Kolyma was 154 km^3^ (with an average interannual run-off rate of 121 km^3^). This fact is not surprising, since it is known that the continental run-off into the Arctic ocean is increasing^16–18^. In August 2017, the average water discharge from the Kolyma was 6552 m^3^/s and in June and July, 21,280 and 12,761 m^3^/s, respectively (Fig. 2). Thus, over 3 summer months, 106 km^3^ of freshwater, 70% of the total run-off, entered the water area of the ESS from the Kolyma River. The greatest influence of the Kolyma run-off was noted at the station 5620 closest to the mouth, where the At/S ratio is 0.089 (Fig. 1c). The influence of the river run-off can be traced up to 150 km away from the southern site, which is 12 nautical miles from the river mouth. With distance from the coast, the influence of the river run-off noticeably decreases, and the ratio does not exceed 0.071, but this clearly shows the presence of freshwater in the entire water column along the transect. In the southern part of the section, between stations 5617 and 5618, a vertical frontal zone is expressed, which is presented as a marginal filter, where the maximum sedimentation of suspended allochthonous matter occurs^19^. The entire water column along the section is undersaturated with aragonite, and in the area of the maximum impact of the Kolyma run-off, *Ω*_*Ar*_ is 0.4–0.6 (Fig. 1d). The vertical front is clearly pronounced, then it increases in the seaward part and reaches 0.95 at the northernmost station of the section 5612 in the 20 m layer. At stations 5612 and 5613, as in the case of the Indigirsky section, the upwelling of bottom waters with a lower *Ω*_*Ar*_ is observed.

### Environmental variables

**Figure 3:**
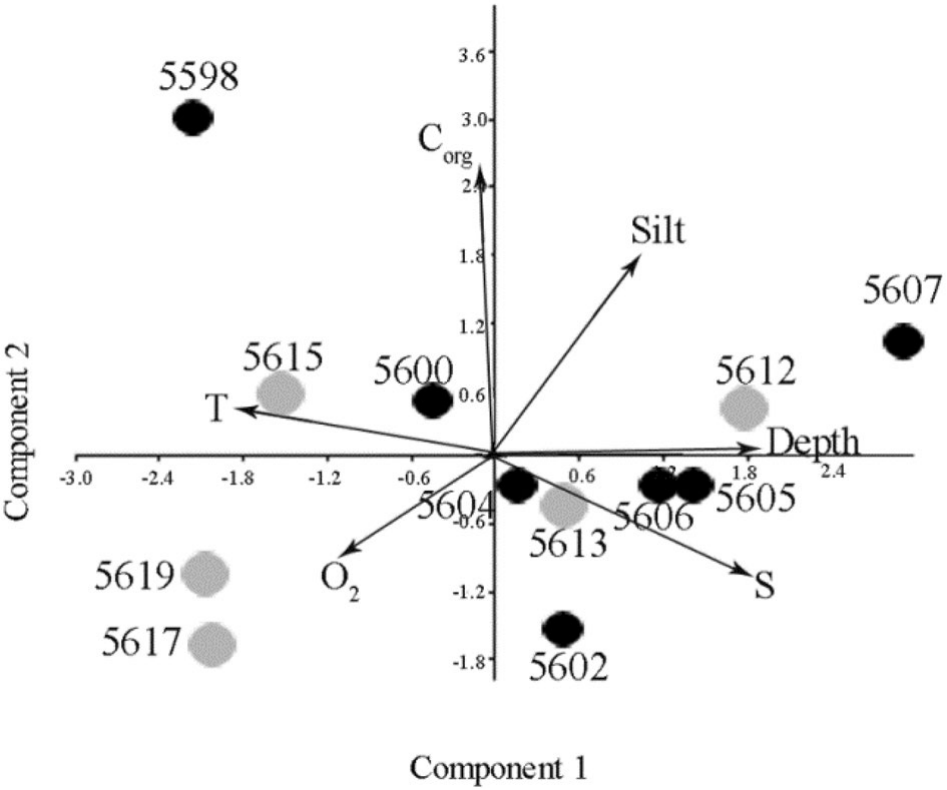
Principal component analysis based on the environmental factors (Depth, S—salinity, T—temperature, O_2_—oxygen saturation, Silt—silt content, C_org_—total organic carbon content). PC1 explained 48% of total variation, PC2—27%. Black circles indicate stations in Indigirka river, grey circles in Kolyma river.

The PCA based on the environmental data showed that the first component accounted for 48% of the total variation and reflected the general trend along the cross-sections from warm shallow brackish water stations 5598, 5617, and 5619 to deepest cold, saline waters stations 5607 and 5612 (Fig. 3). Station 5615 characterized by highest water temperature (4 °C), while it was located further than southernmost stations. The station 5598 was enriched by the highest total C_org_ among all stations. The second component explained 26% of the total variation and was correlated with total organic carbon. The sediment at all of the stations contained a high volume of silt fraction, more than 81%. Only two southern stations of the Kolyma transect (5617 and 5619) were characterized by coarser sediments. The stations 5607 and 5612 were characterized by low oxygen content.

### Meiobenthos

**Figure 4:**
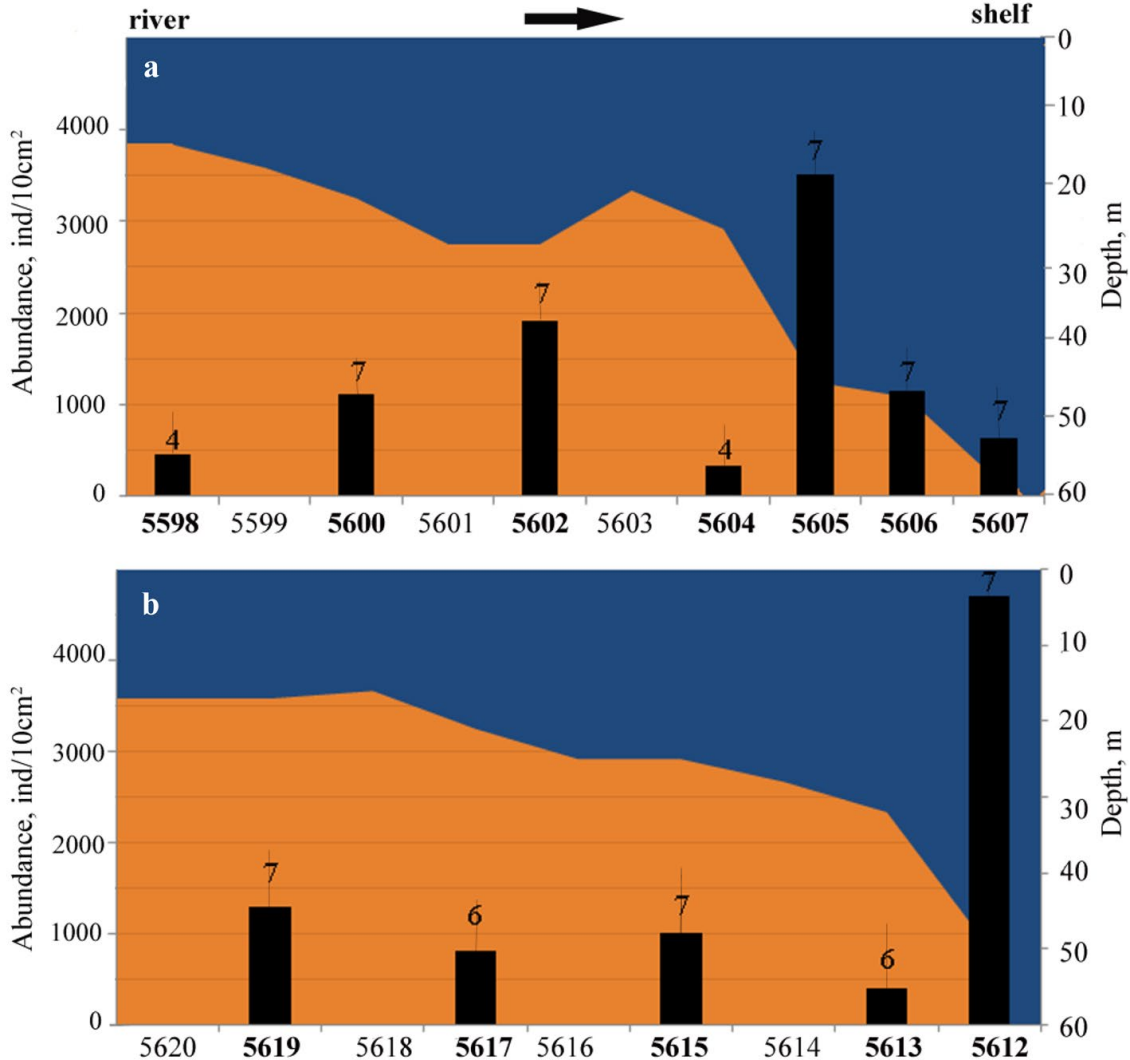
Distribution of abudance of total meiobenthos with depth along Indigirka (**a**) and Kolyma (**b**) transects. Number of meiobenthos taxa recorded for each station is given above columns indicating abundances. Meiobenthos stations marked by bolt. The sea bottom profile is shown in orange.

At the Indigirka transect, the meiobenthic density increased from the southernmost station to the shelf and decreased again at the deepest station (Fig. 4a).

**Figure 5:**
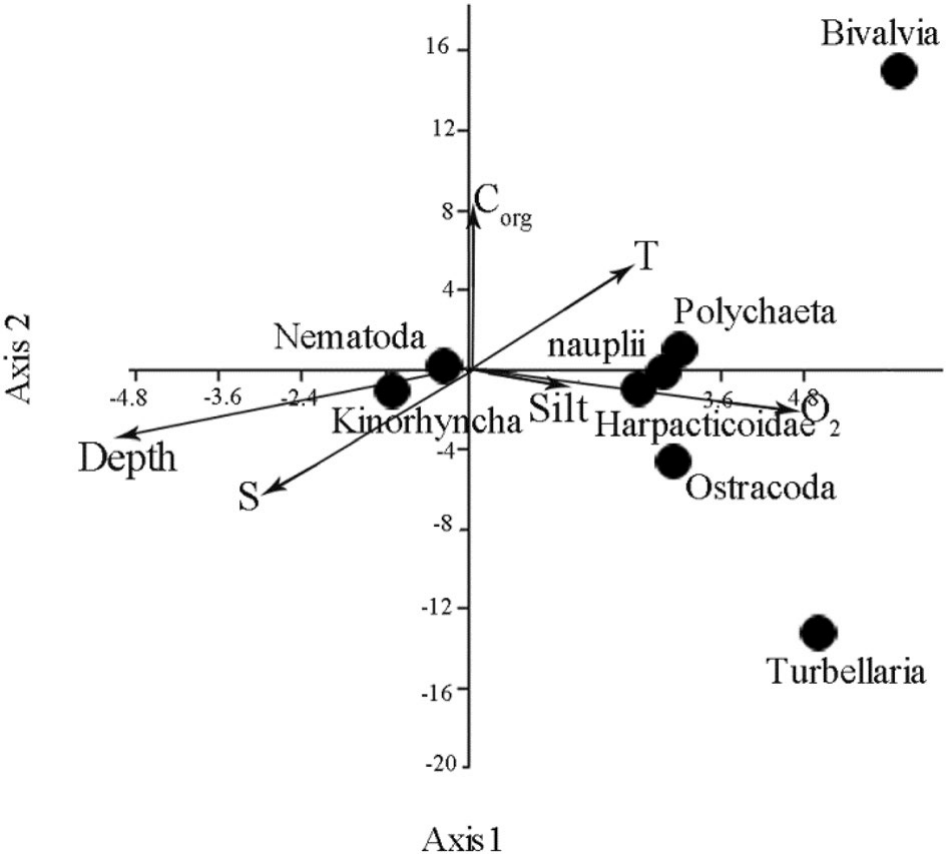
Canonical correspondence analysis showing abundance distribution of meiobenthic taxa in relation to environmental variables: Depth—depth, S—salinity, T—temperature, O_2_—percentage of oxygen, C_org_—total C org, Silt—silt content.

Meiobenthic total densities reached a maximum of 3504 ± 1030 (SE) ind./10 cm^2^ at station 5605. The minimum meiobenthos were noted at stations 5598 and 5604 (465 ± 129 (SE) ind./10 cm^2^ and 334 ± 80 (SE) ind./10 cm^2^, respectively). At the Kolyma transect was observed different tendencies: high meiobenthic total densities along the transect with a significant drop at station 5613 (406 ± 90 (SE) ind./10 cm^2^) and an increase at the deepest station 5612 (4718 ± 1494 (SE) ind./10 cm^2^) (Fig. 4b). One-way ANOVA comparison of total density among stations did not show any significant difference among all stations (p > 0.05). A Tukey’s comparison of total density revealed significant differences among stations 5605 and other stations, except 5602, also abundance at station 5612 significantly higher than at all other stations (p < 0.001), except 5605. In total, 8 major taxonomic groups, 6 of the permanent meiobenthos and 2 of the temporary meiobenthos were found, excluding copepod nauplii (Table 1). Diversity was 6–7 taxa at all stations except stations 5598 and 5604 with 4 taxa per station (Fig. 4a). At all stations, nematodes, harpacticoid copepods and polychaetas were observed, whereas kinorhynchs came as second taxon at station 5612 and was very abundant at station 5605 (Table 1). Turbellaria was noted only at station 5602. The density of nematodes ranged from 244 ± 18 ind./10 cm^2^ (station 5604) to 4508 ± 347 ind./10 cm^2^ (station 5612). Nematodes represented from 69 to 96% of the total meiofaunal abundance. Harpacticoids were the second most important group. The total harpacticoid abundance was relatively low, on average 42 ± 17 ind./10 cm^2^. The lowest density was found at station 5598 (25 ind./10 cm^2^), and the highest at station 5602 (208 ind./10 cm^2^). The accounted for 67% of the total variability and reflected the general trend along transects from the shallower southern to cold deep stations. The CCA analysis (Fig. 5) showed bivalves were positively correlated with water temperature, but negatively correlated with depth and salinity. The harpacticoid copepods with naulii, ostracods, and polychaetes preferred high values of oxygen and low content of organic carbon in sediments. Kinorhynchs revealed positive correlations with depth and avoided warm waters (Fig. 5). Overall, water temperature and the total organic carbon (19% of the total variability) appeared to be important variables explaining spatial variations in abundance of the meiofauna taxa in the studied transects.

**Table 1:** Mean average density (AD ± SE ind./10 cm^2^) of each meiobenthic group in the 0- to 5-cm deep sediment layer.

Station	Indigirka							Kolyma				
	5598	5600	5602	5604	5605	5606	5607	5619	5617	5615	5613	5612
Nematoda	396	938	1497	244	3133	860	539	1039	716	718	281	4508
Harpacticoida copepoda	25	86	208	53	125	187	49	133	57	132	65	67
Kinorhyncha	0	8	33	3	69	43	8	15	4	30	13	94
Ostracoda	0	8	19	0	18	2	2	19	3	16	15	1
Polychaeta	3	12	18	1	3	3	4	8	2	5	1	7
Bivalvia juv	18	7	0	0	4	1	1	5	11	6	2	2
Hydrozoa	0	6	3	0	46	5	12	16	0	4	0	13
Turbellaria	0	0	2	0	0	0	0	0	0	0	0	0
nauplii	23	50	130	33	106	49	29	58	21	89	29	26
AD ± SE	465 ± 129	1115 ± 306	1910 ± 487	334 ± 79	3504 ± 1029	1150 ± 281	644 ± 176	1293 ± 338	814 ± 235	1000 ± 232	406 ± 90	4718 ± 1494
Taxa	4	7	7	4	7	7	7	7	6	7	6	7

Nauplii were counted for total meiobenthos density, but it was not present a taxon. The stations are listed in the direction from the rivers deltas to offshore.

### Nematode community densities and diversity

**Figure 6:**
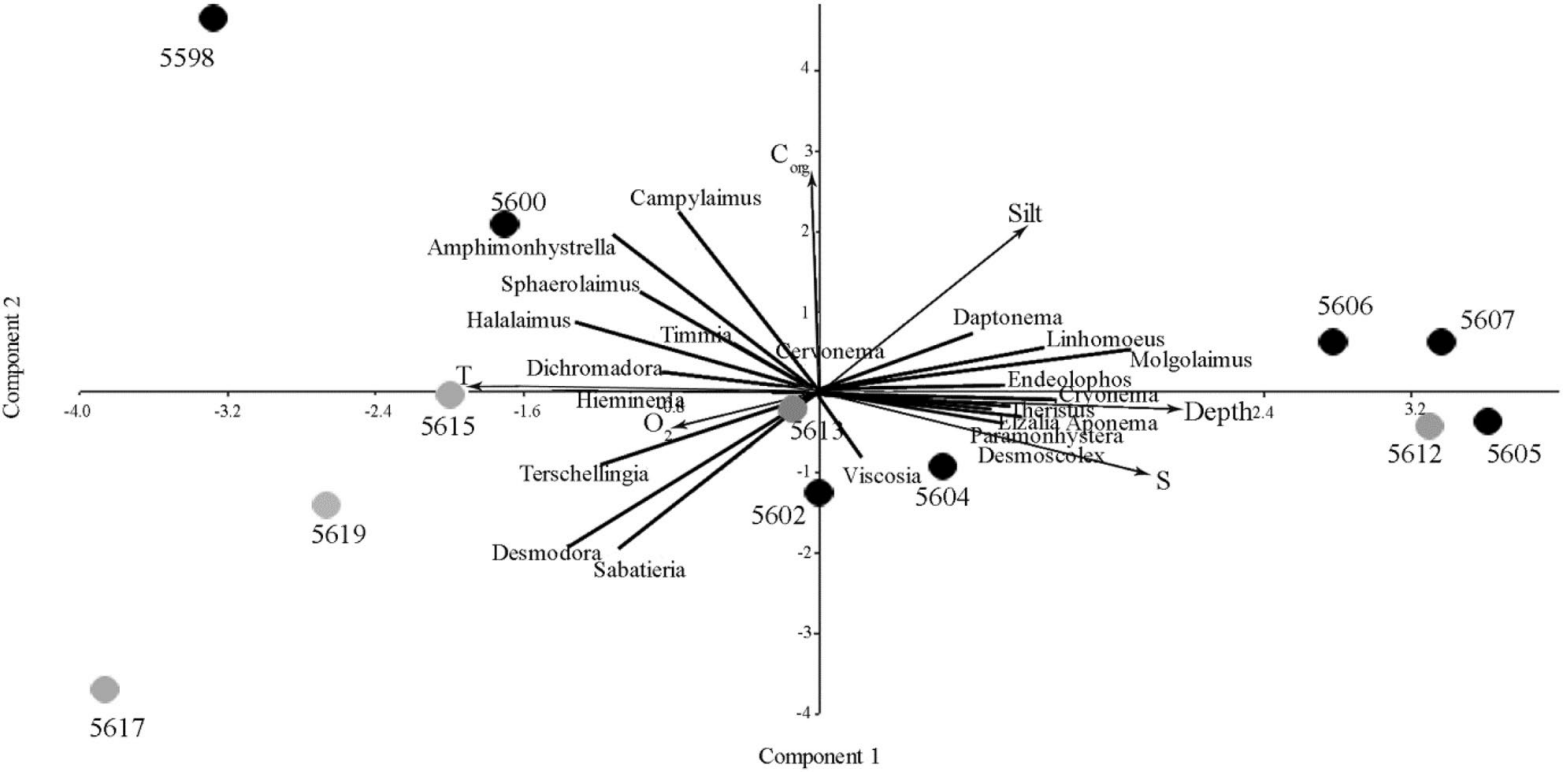
Principal component analysis based on the density of nematodes (genus must be more 1% of total assemblage) and environmental factors (arrows lines: Depth, S—salinity, T—temperature, O_2_—oxygen saturation, Silt—silt content, C_org_—total organic carbon content). Thick lines show the genera of nematodes. Black circles indicate stations in Indigirka river, grey circles in Kolyma river.

At the Indigirka transect, nematode densities increased from the southern shallow water station 5598 (396 ind./10 cm^2^) toward the deeper station 5605 (3133 ind./10 cm^2^). However, below by the shelf, the abundance of nematodes decreased (539 ind./10 cm^2^, station 5607). At the Kolyma transect the opposite trend occurred. The nematode abundance was high at the southernmost station (1039 ind./10 cm^2^, station 5619), and decreased with movement by the shelf to the north. The abundance increased sharply at the deepest and northernmost station (4508 ind./10 cm^2^, station 5612) (Table 1). There were no significant differences between transects, although the mean nematode densities were higher along the Kolyma transect, compared to Indigirka. A total of 1326 nematode individuals were identified from the 12 stations, representing 72 genera belonging to 28 families. The number of genera per station ranged from 15 to 32. The most important nematode families in terms of total density were the Comesomatidae, the Desmodoridae and the Xyalidae, accounting for 14–24%, and the Chromadoridae, the Monhysteridae and the Linhomoidae for 5–11% of the total nematode fauna. The families Xyalidae (9 genera) and Chromadoridae (9 genera) contained the highest number of genera, followed by the Linhomoidae (6 genera) (Table 2). Diversity indices showed no difference in genera composition between both transects. The most different was station 5617, with the lowest genus diversity (15 genera) and strong dominance by two genera, *Sabatieria* and *Desmodora* (Table 2). SIMPER analysis showed that the broadly distributed abundant nematode genera as *Sabatieria, Desmodora, Molgolaimus, Cervonema, Elzalia* and *Daptonema* were primarily responsible for the dissimilarity observed among two transects (Table 3). The multivariate analysis revealed the combined effect of depth and organic content in sediment on nematode community structure at the level of the genera (Fig. 6). The PCA showed that the first component accounted for 26.7% of the total variability and reflected the general along transect trend from shallower warm brackish water (station 5598, 5600, 5617, 5619) to deeper cold salt waters (Fig. 6). The second component accounted for 14.7% of the total variance and correlated with total organic carbon in sediment. The most abundant family Comesomatidae were preferred a more shallow and brackish water environment. There are two important members of this group—*Sabatieria* and *Cervonema* (Fig. 6). The genus *Sabatieria* was sensitive with respect to depth and organic content, and was dominant at warm brackish part of Kolyma transect (5615, 5617, 5619) and in the middle of the Indigirka transect (stations 5602, 5604). The genus *Cervonema* is a clear representative of the more saturated organic content sediments in the brackish waters (Fig. 6). The Desmodoridae were represented by *Desmodora* and *Molgolaimus*. The genus *Desmodora* revealed positive correlation with shallow warm waters at the Kolyma transect as the genus *Molgolaimus* was a clear representative of the deep cold salt waters of the shelf. Amongst one of the most diverse families Linhomoidae, *Terschellingia* was more common in the southern stations in both transects. The Xyalidae are a very diverse family encompassing five important genera: *Amphymonhystrella*, *Daptonema*, *Elzalia*, *Paramonhystera* and *Theristus*. The four genera (*Daptonema*, *Elzalia*, *Paramonhystera, Theristus)* were preferred deep sediments. The genera *Amphymonhystrella, Timmia, Campylaimus, Sphaerolaimus* shows positive correlation with the shallow warm sediments with high content of organic in the Indigirka transect, while genus *Halalaimus* shows the same at both transects. The Chromadoridae are a very diverse group with important genera such as *Dichromadora*, which was more common in the southern warm brackish waters and the genus *Endeolophos*, which was more frequently observed in the deepest part of the Indigirka transect (Fig. 6). The most common sampled genera of Monhysteridae were *Cryonema* and *Hieminema*. These genera were observed opposite trends at both transects: *Cryonema* was abundant at the deep-sea stations while *Hieminema* at the shallow water southern stations (Fig. 6).

**Table 2:** Percentage abundance of nematode genera, their feeding type (FT) and diversity indexes.

Family	Genus	FT	Indigirka transect							Kolyma transect				
			5598	5600	5602	5604	5605	5606	5607	5619	5617	5615	5613	5612
Aegialoalaimidae	*Aegialoalaimus*	1A	1.08	1.6	0.0	2.8	0.8	0.0	2.3	0.0	0.0	0.0	0.0	1.7
Anoplostomatidae	*Anoplostoma*	2B	0.0	1.6	0.8	0.9	0.0	0.0	0.0	0.0	0.0	0.0	0.0	0.8
Axonolaimidae	*Axonolaimus*	2A	0.0	0.0	0.0	0.0	0.0	0.0	0.7	0.0	0.0	0.0	0.0	0.0
	*Margonema*	2A	0.0	0.0	0.0	0.0	0.0	0.0	0.0	0.0	0.0	0.0	2.2	0.0
Camacolaimidae	*Alaimella*	1A	0.0	0.0	0.0	0.0	0.0	0.0	0.0	0.0	0.0	0.0	2.2	0.8
	*Ionema*	1A	0.0	0.0	0.0	0.0	0.0	0.0	0.0	0.6	0.0	0.0	0.0	0.0
Ceramonematodae	*Pselionema*	1A	1.08	0.0	0.0	0.0	0.0	0.0	4.6	0.0	0.0	1	0.0	0.0
Chromadoridae	*Acantholaimus*	2A	0.0	0.0	0.0	0.0	0.8	0.0	0.0	0.0	0.0	0.0	0.0	0.0
	*Chromadora*	2A	1.08	1	0.0	0.9	0.0	3	3.1	0.0	0.0	0.0	2.2	0.0
	*Dichromadora*	2A	3.2	4.1	2.6	2.8	0.8	0.0	0.7	0.0	1.9	15.4	0.0	0.8
	*Endeolophos*	2A	1.08	0.8	1.7	10.5	1.7	24	7	3.4	0.0	2.2	2.2	1.7
	*Euchromadora*	2A	1.08	0.8	0.0	0.0	0.0	0.0	0.0	0.0	0.0	0.0	0.0	0.0
	*Innocuonema*	2A	1.08	0.0	0.0	0.0	0.0	0.0	0.0	0.0	0.0	0.0	0.0	0.0
	*Neochromadora*	2A	0.0	0.0	0.0	0.0	0.0	0.0	0.0	0.0	1.3	0.0	0.0	0.0
	*Timmia*	2A	4.3	5.7	4.3	3.8	0.0	5.2	0.0	4	0.0	0.0	11.4	0.0
	*Trochamus*	2A	1.08	0.8	1.7	0.0	1.7	0.0	1.5	0.0	0.0	0.0	0.0	0.0
Comesomatidae	*Cervonema*	1A	2.2	18	0.8	1.9	11	9	1.5	1.3	4.5	13.2	2.3	0.0
	*Comesomoides*	1B	0.0	0.0	0.0	0.0	0.0	0.0	0.7	0.0	0.0	0.0	0.0	0.0
	*Sabatieria*	1B	7.6	13	28.4	17	11.5	2	15.5	21	46.2	29.7	0.0	8.7
Cyatholaimidae	*Cyatholaimus*	1B	0.0	0.0	1.7	0.9	0.0	0.0	0.7	2	0.0	0.0	0.0	0.0
	*Nannolaimoides*	2A	0.0	1.6	0.0	0.0	0.0	0.0	0.0	0.0	0.0	0.0	0.0	0.0
	*Pomponema*	2A	0.0	0.8	0.0	0.0	0.0	0.0	0.0	0.0	0.0	0.0	0.0	0.0
Desmodoridae	*Desmodora*	2A	1.08	0.8	14.6	0.0	0.0	0.0	0.0	23.6	21.8	10	25.0	0.8
	*Molgolaimus*	1B	5.4	3.3	3.4	0.0	10.6	25.0	17	0.0	0.6	1	9	19.3
Desmoscolecidae	*Desmoscolex*	1A	1.08	0.0	2.5	0.0	3.5	1	0.7	0.0	0.6	1	0.0	1.7
Diplopeltidae	*Campylaimus*	1A	10.9	2.4	0.8	0.0	0.8	0.0	0.0	0.6	0.0	0.0	0.0	1.7
	*Southerniella*	1A	0.0	0.0	0.0	0.0	0.0	0.0	0.0	0.0	0.0	0.0	2.2	0.0
Diplopeltoididae	*Diplopeltoides*	1A	0.0	1.6	0.9	0.0	0.8	0.0	0.0	0.0	0.0	0.0	0.0	0.0
Enchelidiidae	*Abelbolla*	2A	0.0	0.0	0.0	0.9	0.0	0.0	0.0	0.6	0.0	0.0	2.2	0.0
	*Symplocostoma*	2B	0.0	0.0	0.9	0.0	0.0	0.0	0.0	0.0	0.0	0.0	0.0	0.0
Enoplidae	*Enoplus*	2B	0.0	0.0	0.0	0.0	0.0	0.0	0.0	0.6	0.0	0.0	0.0	0.0
Leptolaimidae	*Leptolaimus*	1B	2.2	2.5	1.7	0.0	0.0	1	0.0	1.3	0.0	0.0	0.0	0.0
Leptosomatidae	*Leptosomatum*	1A	0.0	0.0	0.0	0.0	0.0	0.0	0.0	1.3	0.0	0.0	0.0	0.0
Linhomoeidae	*Anticyathus*	1A	0.0	0.0	0.0	0.0	0.0	0.0	0.0	0.0	0.0	0.0	0.0	7
	*Desmolaimus*	1B	1.08	0.8	0.0	0.0	0.0	0.0	0.0	0.0	0.0	1	0.0	0.0
	*Eleutherolaimus*	1A	0.0	1.6	0.0	0.0	0.8	0.0	0.0	0.0	0.0	0.0	0.0	0.0
	*Linhomoeus*	1A	0.0	2.5	0.0	0.92	4.4	2	2.3	0.0	0.0	0.0	0.0	0.0
	*Metalinhomoeus*	1B	0.0	0.0	0.0	0.9	0.0	0.0	0.0	0.0	0.0	0.0	0.0	0.0
	*Terschellingia*	1B	5.4	5.7	0.0	1.9	0.0	0.0	1.5	0.0	14	0.0	0.0	0.0
Microlaimidae	*Aponema*	2A	0.0	1.6	0.0	2.8	16	1	0.7	2	0.0	1	0.0	7.9
	*Microlaimus*	2A	2.2	0.0	0.0	0.9	0.8	0.0	1.5	0.0	0.0	3.3	0.0	3.5
Monhysteridae	*Cryonema*	1B	3.3	0.0	0.9	14.2	3.5	5.2	16.3	6.7	0.0	0.0	6.8	15
	*Hieminema*	1B	7.6	0.0	0.9	8.5	0.8	1	0.7	12.2	0.0	1	0.0	0.0
	*Monhystera*	1B	0.0	0.8	0.0	0.0	0.0	0.0	0.0	0.0	0.0	0.0	0.0	0.0
Monopothiidae	*Monoposthia*	2A	0.0	0.0	0.0	0.0	0.0	0.0	0.0	0.6	0.0	0.0	0.0	0.0
	*Nudora*	2A	0.0	0.0	0.9	0.0	0.0	0.0	0.0	5.4	0.0	0.0	0.0	0.0
Oncholaimidae	*Metoncholaimus*	2B	0.0	0.0	0.0	0.99	0.0	0.0	0.0	0.0	0.0	0.0	0.0	0.0
	*Oncholaimellus*	2B	0.0	0.0	0.0	0.0	0.0	0.0	0.0	0.0	0.0	0.0	2.2	0.0
	*Viscosia*	2B	0.0	0.0	2.6	8.6	0.8	0.0	0.0	0.0	0.6	0.0	2.2	0.8
Oxystominidae	*Halalaimus*	1B	7.6	3.3	0.0	0.9	0.0	0.0	2.3	0.0	3.8	8.8	0.0	0.8
	*Oxystomina*	1A	0.0	0.0	1.7	1.9	0.0	1	0.7	0.6	0.0	0.0	0.0	0.0
	*Thalassoalaimus*	1B	0.0	0.0	0.0	0.0	0.8	0.0	0.0	0.0	0.0	0.0	0.0	0.0
Rhabdolaimidae	*Syringolaimus*	2A	0.0	0.0	0.0	0.0	0.0	0.0	0.0	0.0	0.0	0.0	2.2	0.0
Selachinematidae	*Latronema*	2B	0.0	0.0	0.0	0.0	0.0	0.0	0.7	0.0	0.0	0.0	0.0	0.0
Sphaerolaimidae	*Metasphaerolaimus*	2B	0.0	0.0	0.9	1.9	0.0	0.0	0.0	0.0	0.0	0.0	0.0	0.0
	*Parasphaerolaimus*	2B	0.0	0.0	0.0	0.0	0.0	0.0	1.5	1.3	0.0	0.0	0.0	0.0
	*Sphaerolaimus*	2B	6.5	6	4.3	0.0	0.8	0.0	2.3	1.3	1.9	0.0	11.4	0.0
	*Subsphaerolaimus*	2B	0.0	0.0	0.0	0.0	0.0	0.0	0.0	0.0	0.6	1	0.0	0.0
Thoracostomapsidae	*Enoploides*	2B	0.0	0.0	0.0	0.0	0.8	0.0	0.0	0.0	0.0	0.0	0.0	0.0
	*Mesacanthion*	2B	0.0	0.0	0.9	0.0	2.6	0.0	0.0	0.7	0.0	0.0	0.0	0.0
	*Paramesacanthion*	2B	0.0	0.0	0.0	0.0	0.0	0.0	0.0	0.0	0.0	1	0.0	0.0
	*Parasaveljevia*	2B	0.0	0.0	0.0	0.0	0.0	0.0	0.0	0.0	0.0	0.0	2.2	0.0
Tripyloididae	*Gairleanema*	2B	0.0	0.0	0.0	0.0	0.0	0.0	0.0	0.0	0.6	0.0	0.0	0.0
Xyalidae	*Amphimonhystrella*	1B	9.8	4.1	1.7	0.0	0.0	1	0.7	3.4	0.0	0.0	2.2	0.0
	*Daptonema*	1B	6.5	0.8	7	3.8	3.5	12.5	7.7	0.7	0.6	7.7	4.5	0.8
	*Elzalia*	1B	1.08	0.8	6	1.9	1.7	1	2.3	0.0	0.0	0.0	2.2	24.6
	*Filipjeva*	1B	1.08	3.3	0.0	0.9	0.0	1	0.7	0.0	0.0	0.0	0.0	0.0
	*Gnomoxyala*	1B	0.0	1.6	0.0	0.0	0.8	0.0	0.0	0.0	0.0	0.0	0.0	0.0
	*Gonionchus*	2B	0.0	0.0	0.0	1.9	0.0	0.0	0.0	0.6	0.0	0.0	0.0	0.0
	*Linhystera*	1A	0.0	0.0	0.0	0.0	0.0	0.0	0.0	0.6	0.0	0.0	0.0	0.0
	*Paramonhystera*	1B	2.2	1.6	5.2	1.9	3.5	1	0.7	0.6	0.6	1	2.2	0.0
	*Theristus*	1B	0.0	3.3	0.0	1.9	12.3	2	0.0	2	0.0	0.0	0.0	0.8
	S		28	32	27	28	27	19	29	27	15	17	20	19
	Dominance		0.06	0.07	0.12	0.08	0.08	0.15	0.09	0.12	0.28	0.15	0.11	0.14
	Simpson_1-D		0.93	0.92	0.87	0.91	0.91	0.85	0.9	0.87	0.71	0.84	0.88	0.85
	Shannon_H		3.01	3.03	2.65	2.84	2.75	2.26	2.73	2.52	1.66	2.19	2.59	2.28
	Pielou index		0.90	0.87	0.80	0.85	0.83	0.76	0.81	0.76	0.61	0.77	0.86	0.77

Stations are indicated in the top line in bold. Number of genera—S.

**Table 3:** Fifteen most important nematodes genera in the ESS.

Taxon	Av. dissim	Contrib. %	Cum %	Indigirka MA	Kolyma MA
*Sabatieria*	9.606	14.21	14.21	15.6	28
*Desmodora*	7.277	10.77	24.98	2.71	18
*Molgolaimus*	4.495	6.65	31.62	10.1	5.6
*Cryonema*	3.457	5.114	36.74	7	6
*Cervonema*	3.11	4.601	41.34	7.29	4.4
*Elzalia*	3.07	4.541	45.88	2.43	5.8
*Endeolophos*	2.903	4.294	50.18	7	2
*Terschellingia*	2.388	3.533	53.71	2.29	4.4
*Hieminema*	2.27	3.359	57.07	2.86	3.8
*Daptonema*	2.253	3.334	60.4	6.43	2.4
*Aponema*	2.035	3.011	63.41	3.57	2.6
*Dichromadora*	1.877	2.777	66.19	2.29	3.6
*Halalaimus*	1.527	2.259	68.45	2.14	3
*Theristus*	1.464	2.166	70.61	3.14	0.8
*Timmia*	1.409	2.084	72.7	3.57	2.2

Av. dissim.—overall average dissimilarity; Contrib.—contribution (%); Cum—cumulative (%); MA—mean abundance in each of the two transects. Indigirka—Indigirka transect, Kolyma—Kolyma transect.

### Nematode feeding types

**Figure 7:**
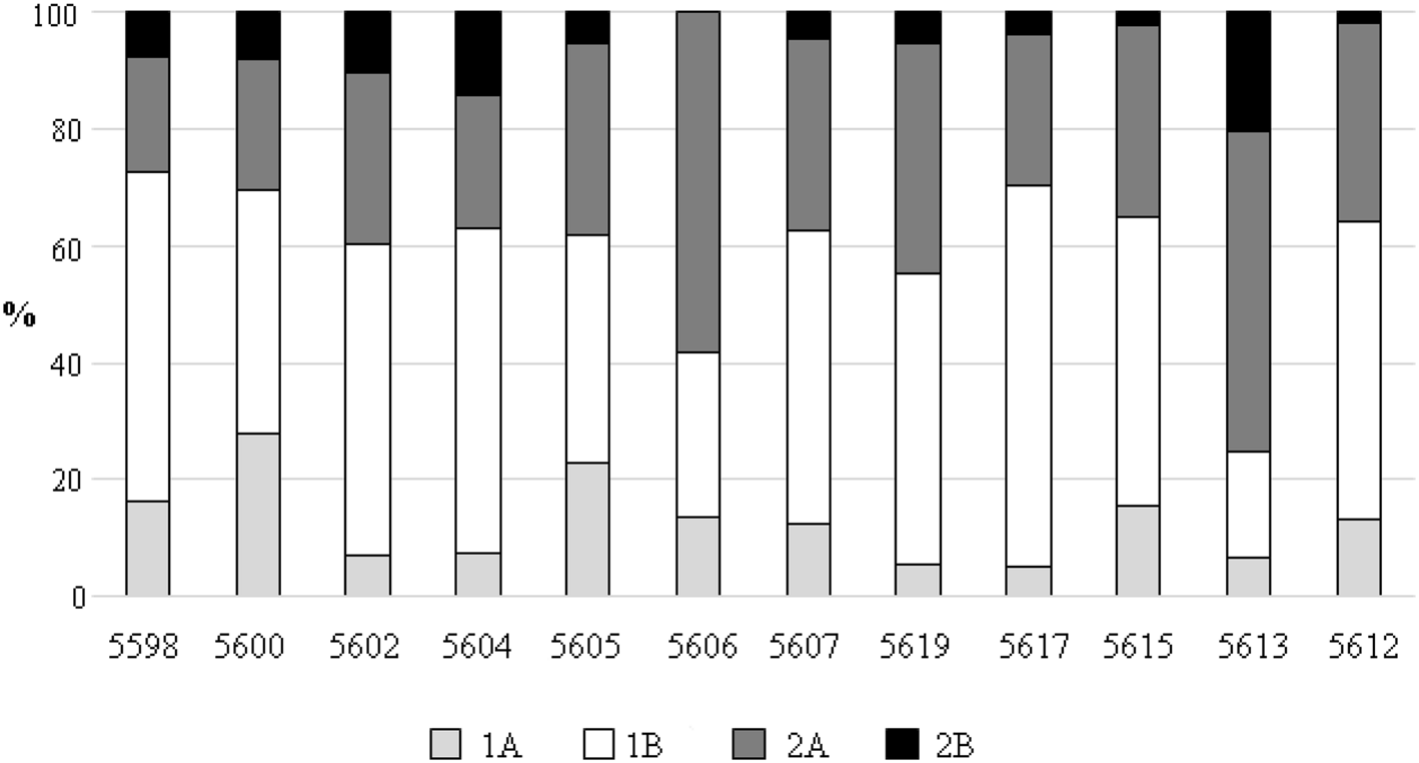
Percentage of nematode trophic groups revealed in the study area.. 1A—selective deposit feeders, 1B—nonselective deposit feeders; 2A—epistrate feeders; 2B—predators.

The analysis of the feeding types showed a significant dominance of nonselective deposit feeders (1B feeding type) and epistrate feeders with a small stoma supplied with teeth, stilets and dentacules (2A feeding type). Nematodes with an unarmed or very narrow stoma, without teeth (1A feeding type) made up 5–28% of the total nematodes in all stations. The selective deposit feeders (1A) were more abundant in silty sediments with organic enriched, whereas depth doesn’t have much of an impact on them. The most abundant nematodes with 1A type were *Campylaimus* and *Cervonema*. Non-selective deposit feeders made up more than 39% of all nematodes at all stations except 5606 and 5613 (Fig. 7). At these stations, epistrate feeders were dominant (58 and 55% correspondently). The most numerous non-selective deposit feeders (1B, *Sabatieria*) and epistrate feeders (2A, *Desmodora*) show negative correlation with organic content and silt, however 1B prefer more coarser sediment (5617, 5619). The highest percentages of species belonging to scavengers and predators (2B feeding group) with massive teeth or armature in the buccal cavity were found at stations 5604 and 5613 (14–20%). This group of nematodes was almost absent at station 5606. No clear trend was observed for the distribution of feeding group 2B. The most abundant predators were *Sphaerolaimus* and *Viscosia* (Fig. 6). The analysis of the contribution of feeding types to similarity within each group suggested that there were broadly distributed feeding types such as selective deposit and epistrate feeders. The dissimilarity among the Indigirka and Kolyma was caused by differences in the density of the selective feeders and predators (Table 4).

**Table 4:** Feeding types of nematodes in the East Siberian Sea.

Feeding type	Av. dissim	Contrib. %	Cumulative %	Indigirka	Kolyma
1B	3.945	30	30	51.3	57.4
2A	1.91	14.53	61.61	34	38.2
1A	1.317	10.02	71.62	17.1	9.6
2B	0.7	5.323	93.02	8	5.4

Comparison feeding types between two transects—Indigirka and Kolyma.

## Discussion

Generally, there are major structuring factors—such as salinity, physical–chemical properties of the sediment, food availability—that play the most important roles in the meiobenthic distribution patterns (Moens et al.^20^). However, the relative significance of the various factors can differ considerably among habitats and regions^9,21,22^ (Alves et al.^29^). The Arctic Ocean is the most riverine-influenced of all the world’s oceans, with freshwater inputs great enough to create an estuarine-like environment throughout the ocean basin that is characterized by low-salinity surface waters with a clear terrigenous chemical signal^23^. Given that the Arctic seas flow with large rivers, the Ob, the Yenisey, the Lena, the Indigirka, and the Kolyma, one of the main structuring factors influencing the meiobenthos is salinity in the estuarine-shelf systems^10,14^. The maximum impact of the Indigirka run-off was at southernmost station 5598 with warm water temperatures and the highest organic carbon content and low density and diversty of meiobenthos, while the strong signal of the Kolyma waters was traced at a distance of 200 km from the beginning of the transect with the maximum at the southern station 5619 and high abundance of meiofauna. Comparing the influence of a huge river as the Amazon with Indigirka and Kolyma rivers, on the inner shelf meiofauna, observed the similarity—in the nearshore sites is lower meiofaunal and nematodes abundance than father offshore shelf sediments. The nearshore deposits of the Amazon subaqueous delta complex generally have a surface layer of fluid mud and are devoid of macrobenthos and characterised by low density of meiofauna. The high degree of physical disturbance and unstable nature of the seabed coupled with reduced detrital food availability in bottom sediments were the major factors limiting faunal abundances, controlling the taxonomic diversity of the meiobenthos^21^. Earlier studies in ESS noted that the low meiobenthos abundance in the close to the shore was caused by shallow depths, desalination, high turbidity, and the abrasive action of the ice^3,5,24^. It can be assumed that in the zone of active mixing with shallow depths, desalination and an increase in water temperature, meiobenthos does not feel comfortable, while with the removal of the shelf towards marine conditions, the abundance of meiofauna begins to increase. However, in addition to harsh conditions in the zone of turbulence the finer sediments may facilitate burrowing, overcome movement barriers, sufficient oxygen saturation of sediment thus provide more suitable habitat for meiobenthos. Sediment grain size is one of the main factors related to the abundance and composition of the meiobenthos, and driving important deviations from the general trend shaped by salinity^14,25–29^. Significant change in both the abundance and species composition of meiobenthos taxa was revealed, depending on the grain size type in the Kara Sea^30^. Recent studies in the Chukchi Sea have shown a lack of sediment influence in shaping meiofauna distribution^31^. In the ESS the sediment was silty at all stations, except for two stations (5617, 5619) with coarse grain content. The granulometric composition and low values of organic content appear to influence the meiobenthos abundance inhabited in the nearshore zone under the influence of the Kolyma River. Numerous studies have documented the influence of organic matter on meiofauna^32^. However, it has been reported that high levels of organic matter or pollution both reduce and increase the density and diversity of the meiofauna^9,29^. The highest values (> 80%) of terrigenous organic matter in the ESS sediments were found in the nearshore areas of the Indigirka and the Kolyma rivers^33^. More than three-fold excess of Pb and high rates of accumulation of Cr, Co, Ni, Cu and Zn were detected at the mouth of the Indigirka River at station 5598^34^. The mesocosm experiment showed high rates of Pb to affect meiofaunal community by reducing the richness or diversity, and the abundance of the most sensitive taxa^35^. It can be assumed that the low values of abundance and diversity of meiofauna at the southernmost Indigirka transect station are caused by the influence of both terrigenous drift and high content of heavy metals in the sediment. The high concentration of phosphates, low nutrients and Si, and low oxygen indicated an area at the slope (station 5604) with downwelling of cold sea waters and orographic depression with the accumulation of organic matter. Maximum of ammonia nitrogen and total mineral phosphorus in the bottom layer due to location at the depression with accumulation of sedimentation of the riverine suspended particulate matter was observed at station 5604. The low oxygen saturation of the water combined with the high content of ammonium nitrogen indicated the predominance of degradation processes of organic matter and the absence of the active phase of photosynthesis. The high concentration of nitrite nitrogen, ammonium nitrogen and phosphorus indicates a recently started destruction process and may be the reason for the decrease in the density of meiofauna at station 5613. All taxa of meiobenthos were depressed at these stations. The low near-bottom oxygen content was due to the oxidation of organic matter in the bottom waters and the upper layer of sediment. Both areas of reduced oxygen content were confined to places with suspension accumulation (the foot of the slope in the northern part of the transect and a local depression in the central part). One of the stations was located at the marginal ice zone. Marginal ice zones are known to be the most highly productive systems in the Arctic Ocean with large amounts of primary production reaching the deep seafloor^36^. At station 5607 there was the high diversity of meiobenthos and nematode community, while the density was low. Presumably, the reduced abundance of meiofauna at station 5607 was related to its low oxygen content (60.5%), which in turn was associated with melting ice in the marginal ice zone. The general distributions of water hydrochemical data and nitrates at the ESS better correspond to the late autumn. The oxidation processes of organic matter in the water column reduced the food flow for the meiobenthos.

The benthos of the ESS shelf has repeatedly been reported to be generally poorer than in other Eurasian-Arctic seas in terms of diversity, abundance and biomass^4,6^. This scarcity has been attributed to the severe climate in the ESS, as well as to the features of the basin’s hydrology^6^. A comparison of the meiobenthos abundance among the East Siberian, the Laptev and the Kara seas shows a high variability of density on the Arctic shelf, due to a variety of hydrological and hydrochemical conditions^10,37^. The analysis of the meiobenthos taxonomic composition on the Kara Sea shelf showed 15 meiofauna taxa, 14 taxa in the Laptev and Chukchi Seas^10,15,31,37,38^. Six major taxa of permanent and two of temporal meiobenthos were identified in the present study, which confirms the relatively low diversity of the meiofauna at the western part of the ESS. Nematodes have always been the most abundant, harpacticoids have been the second group, but kinorhynchs have come as second or third taxon. The dominance of nematodes in the meiofauna is a feature of the estuarine shelf zone not only of the Arctic seas. Nematodes have dominated in the meiobenthos communities inhabiting the northeastern Brazil continental shelf affected by the vast Amazonian drainage complex and continental shelf near Changjiang (Yanngtze)^21^. Densities of kinorhynchs displayed differences between stations, with the highest values at the stations with maximum abundance of nematodes and the lowest at stations located at the south of transects. A relatively large number of kinorhynchs were detected at a few stations and formed the second or third most dominant taxon at some stations in the Chukchi Sea^31^. Little is known about the main environmental factors that shape the kinorhynch communities in general. The recent study performed in the Arctic Ocean determined that sediment grain size is the variable that most seem to affect the Kinorhyncha species composition^39^. The study sites located at the mouth flow of Mobile Bay with outflow of freshwater into the Gulf of Mexico has revealed that sediment type is likely the main driver affecting kinorhynch abundances and diversity^40^. However, based on the results obtained, it is impossible to draw conclusions about the influence of any factors on the distribution of kinorhynchs.

In the study area, the most abundant families and genera of nematodes were the representatives of the assemblages of Arctic sediments^10,15^ and they may be considered as typical iso-communities of muddy sediments (Heip et al.^67^). Communities of nematodes were quite similar along the Indigirka transect, but the closest in composition were in the sediments at the terminal stations. In contradiction of desalination, a significant difference in depth (13 *versus* 59 m), high organic content and a noticeable deficiency of oxygen, the nematode population density decreased without changing the composition of the community. The southernmost station was different with the dominant genus *Campylaimus* in the nematode community. The genus *Campylaimus* is a broadly distributed but relatively uncommon genus of marine and brackish nematodes with 20 nominal species and one *nomen nudum*^41^. Many species descriptions and redescriptions are based on a very few (single) individuals, which limits understanding of inter- and intra-specific variability and morphology-based species boundaries^41^. Specimens from the ESS are represented only by females, that is why the identification to the species level would be unreliable and approximate. The deepest and the most distant site was also inhabited by a diverse community of nematodes with three numerous genera: *Sabatieria*, *Molgolaimus* and *Cryonema*. The most interesting fact is the high density of the *Cryonema*, which we assume is due to the station's location on the marginal ice zone. Ice nematodes were represented by juveniles and females that could colonize the sediment from melting ice. The sediment at the orographic depression was inhabited by a highly diverse nematode community despite nematodes being decreased in abundance. The dominant genus was *Sabatieria*, which generally shows a high tolerance to hypoxic conditions (Broman et al.^45^). It should be noted that this genus was the most widespread in the entire studied area. The Comesomatidae with the genus *Sabatieria* was the dominant family at the Chukchi Sea slope at depths 898–1945 m^42^. A higher abundance of *Sabatieria* at the shallow slope was described for the Kara and the Laptev Sea shelf sites, and the estuarine sites the NE Atlantic, the Baltic Sea, and the Black Sea^43^ (^45,46^).

At the Kolyma transect there is an opposite trend. The nematode abundance is high at the southernmost station (1039 ind./10 cm^2^, station 5619), and decreases with movement to the shelf to the north. The abundance increases sharply at the deepest and northernmost station (4508 ind./10 cm^2^, station 5612). The genus *Desmodora* is widespread, one of the most adaptable, inhabiting sediment both in freshwater and in the sea, and it was abundant at shallow brackish water sites, including station 5613^44,47^. At this site there was impoverishment of the nematode assemblage, primarily the density of marine species and the capability of surviving in brackish water species. The station 5612 was deep with silty sediments, situated further north along the transect, influenced by cold, euhaline (32 psu) waters with a low diversity of macrobenthos (Kokarev, in preparation). The concentration of bacterioplankton at station 5612 increased with depth, and reached maximum values in the bottom layer^48^. Perhaps all of these conditions achieved a positive effect on the nematode community.

The distribution of food from the surface waters is a major driver for the aggregated distribution pattern of nematode abundance and reproduction events^11^. Sediments with high silt content generally show a high proportion of deposit feeders (Heip et al.^67^). The epigrowth feeders (2A) and non-selective deposit feeders (1B) were abundant in the western part of the East Siberian Sea shelf, while they were represented by low numbers in deep-sea meiofauna studies^42,49^. This is probably related to the sedimentation of organic matter and a sufficient amount of bacteria in the shallow water shelf. Predators did occur in low numbers in the orographic depressions where decay processes occur, which negatively affected the nematode community. Most predators and omnivores are also facultative scavengers and this combination of feeding types is a successful feeding strategy in food-limited habitats such as the deep sea or a bottom depression^50^.

## Materials and methods

### Study area

The ESS is one of the marginal and most ice‐bound seas off the Arctic Ocean bordering the eastern part with the Chukchi Sea in the east by the De Long Strait and with the Laptev Sea in the west via the Dmitry Laptev, Eterikan and Sannikov Straits. More than 70% of the sea bottom area comprises depths of less than 50 m^51^. Two major rivers enter directly into the ESS the Indigirka (152° E) and Kolyma (162° E) rivers. The mean annual run-off of the Kolyma River (121 km^3^/year) exceeds that of the Indigirka (50 km^3^/year) by almost twofold and discharge during the high-water period may increase tenfold^52^ (Fig. 2).

Based on the distribution of the hydrological and hydrochemical data, the ESS is separated into two regions, western and eastern. The western area is affected by freshwater input by Laptev Sea water masses. Atlantic water entering in the western regions of the ESS is heavily diluted by the Lena River, even before entering the ESS and by the Indigirka and Kolyma rivers run-off and the freshwater spatial distribution is largely dependent on the wind^53^. The eastern area experiences Pacific influence. The long-term average position of the frontal zone separating these two regimes is around 160° E for the water column and 170° E for the sediments^33^.

**Figure 8:**
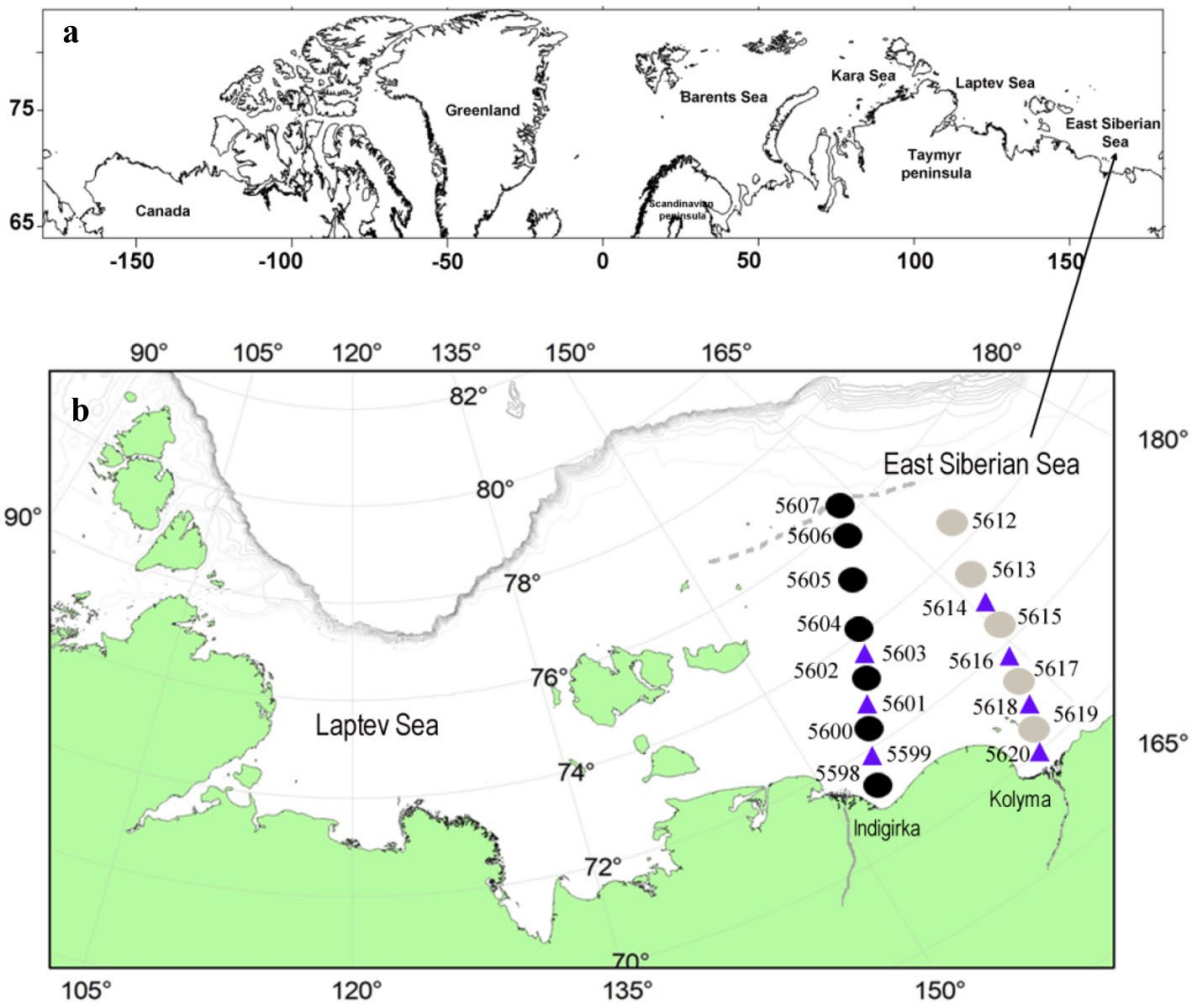
Location of East Siberian Sea (**a**) and map with station positions in the East Siberian Sea (**b**). Map was created with Golden Software Surfer version 8. Black circles indicate stations in Indigirka river, grey circles in Kolyma river. Blue triangles indicate additional hydrochemical stations without meiobenthic sampling. Ice extend during AMK 69 shown by grey dotted line.

The ESS seawater temperature is close to the freezing point over the entire water column during the winter as a result of surface cooling and ice formation, whereas the bottom layer may well be affected by warm Atlantic water coming from the continental slope^54^. The main processes that affect the accumulation of organic matter in the ESS are the terrigenous contribution of river runoff, coastal erosion, and primary production associated with the inflow of Pacific waters^55^. In general, organic carbon in the surface sediments is of terrigenous origin and varies from 0.5 to 1.5% in the ESS and the higher values are associated with sediments near the Indigirka and Kolyma river mouths^56^. Our studies were performed in the near‐shore zone (open water between the coast and drifting ice) of the ESS (Fig. 8). The maps (Fig. 8) were prepared with Surfer software version 8 (http://www.goldensoftware.com).

The sea ice coverage in the autumn of 2017 was low and a large area of the ESS could be sampled. Only some small patches of drifting sea ice were observed between stations 5606 and 5607 during the cruise, but the sea ice boundary at station 5607 restricted the sampling program. The river run-off also impacts the chemical signature of the waters as it has a high concentration of total alkalinity, dissolved organic carbon as well as nutrients (sum.^53^). In 2017, the investigated area was highly influenced by freshwater from the rivers. It should be noted that several sources of freshwater exist in this area: rivers as well as sea ice melt. The high-water period usually occurs in May–June and makes up almost half of the annual run-off, and therefore, by the time the expedition was carried out, most of the Indigirka run-off was already in the water area of the ESS^52^. The surface water salinity exhibited a longitudinal trend characterized by low values (24–27 psu) in the southernmost stations while the sea shelf waters showed relatively higher values (33 psu). The surface water temperature was decreased from south (7 °C) to the north (− 2 °C) due to the influence of river flow in the south and the descent of seasonal ice in the north^48^. The seasonal variation of the physical–chemical parameters, such as the low concentrations of nitrates (0–0.5 µM) and low oxygen saturation (54–96%) corresponded to the end of the autumn/beginning of the winter season^48^.

### Water samples and characteristics

Data on temperature and salinity were measured directly with a CTD-profiler SBE19 plus (SeaBird Electronics, USA). Samplings for hydrochemical data (pH, total alkalinity *At*) were collected in plastic 0.5-L bottles without preservation with a carousel of 12 five-liter plastic bathometers (General Oceanics, USA). Before analysis of total alkalinity, the samples were pre-filtered through 0.45-µm Millipore filters^57^. The pH value (NBS scale) was determined using the potentiometric method (Dickson, Goyet, 1994) on the pH-meter “HANNA HI 2210”, and for calibration, we used the appropriate HANNA buffer solutions. Analysis of total alkalinity was conducted by direct titration (the Bruyevich method) on an “easyTitrino” titration unit (Italy) with a visual determination of the titration end-point^58^, which is comparable with other methods of total alkalinity determination^59,60^. Aragonite saturation (Ω_Ar_) was calculated using temperature, salinity, total alkalinity and pH data with “Program Developed for CO_2_ System Calculations”^61^. We used the *At/salinity (At/S)* ratio as a reliable marker of continental run-off influence on the water structure of the ESS. An *At/s* value of greater than 0.07 units shows the presence of riverine water, while less than 0.07 units is plain seawater. Data on the discharge of the Kolyma and Indigirka Rivers in the entire year of 2017 was obtained from the Arctic Great Rivers Observatory project web-site^62^.

### Sampling and treatment of meiofauna samples

Meiobenthic samples were collected at the East Siberian Sea in September 2017 from aboard the R/V “Akademik Mstislav Keldysh” during the AMK 69 cruise using a Neimistö corer^63^. Samples were collected along the 150° E and 160° E latitudinal transects at water depths from 13 to 59 m from the deltas of the Indigirka and Kolyma Rivers to adjacent parts of the East Siberian Sea shelf (Table 5). The shallowest stations were located near the river deltas while the deepest one was at the shelf. Twelve sediment samples were picked up to study the nematode community, and seven additional stations (5599, 5601, 5603, 5614, 5616, 5618, 5620) were collected for analyzing only hydrochemical characteristics. Concurrent with the sampling, environmental parameters such as the depth, surface and bottom salinity, bottom water temperature in the water column, nutrient (phosphates, silicates, nitrogen, oxygen) concentrations, grain size and C_org_ were measured from the same Neimistö corer sediment sample^48^.

**Table 5:** List of sampling stations, locations, depths, and variables characterizing the near-bottom water and sediments (0–5 cm) (S—salinity, Temp—temperature, O_2_—percentage of oxygen, C_org_—total C org, Silt—% <50 mkm silt content.

	Stations	Latitude (°N)	Longitude (°E)	Depth (m)	Bottom water					Sediment	
					T (°C)	S (psu)	O_2_	PO_4_ µM	NO_2_ µM	Silt	C_org_
Indigirka transect	**5598**	72° 19	151° 431	14	3.0	24.583	82.4	0.97	0.25	100	1.51
	5599	–	–	17	2.87	26.599	86.1	0.85	0.11	–	–
	**5600**	72° 19	154° 31	21	0.6	30.155	87.4	1.08	0.19	99.6	0.90
	5601	–	–	27	− 1.31	32.692	86.0	1.16	0.33	–	–
	**5602**	72° 19	156° 26	27	− 1.4	32.611	87.7	1.26	0.20	94.1	0.45
	5603	–	–	20	1.23	28.222	92.4	1.16	0.05	–	–
	**5604**	74° 5	158° 20	25	− 1.1	31.708	91.6	1.45	0.13	97.6	0.79
	**5605**	74° 52	160° 10	45	− 1.7	32.440	83.8	1.16	0.06	99.9	0.75
	**5606**	75° 38	161° 59	47	− 1.7	31.841	87.1	0.80	0.06	94.7	0.88
	**5607**	76° 10	163° 3	58	− 1.3	33.429	60.5	1.02	0.04	99.9	0.94
Kolyma transect	**5612**	74° 23	168° 11	50	− 1.7	31.550	74.6	1.20	0.06	100	0.79
	**5613**	73° 20	166° 47	32	− 1.2	30.036	86.6	1.48	0.28	99.4	0.57
	5614	–	–	28	1.89	28.393	92.0	1.09	0.08	–	–
	**5615**	164° 20	165° 27	25	4.0	28.208	91.7	1.18	0.04	98.1	0.86
	5616	–	–	25	5.0	28.843	88.7	1.26	0.04	–	–
	**5617**	71° 21	164° 20	21	3.67	29.196	84.4	1.37	0.05	81.1	0.54
	5618	–	–	16	4.82	26.528	83.7	1.32	0.11	–	–
	**5619**	70° 25	163° 04	17	2.23	26.617	79.1	1.33	0.23	88.6	0.46
	5620	–	–	17	0.75	27.762	78.6	1.38	0.15	–	–

Meiobenthos stations are shown in bold.

At the meiobenthic stations, we also took into account the % of silt fraction (particle size < 0.05 mm) and % of total organic carbon in the upper 5 cm of the sediment. Sediment samples for particle size determination and organic matter content were collected at the same stations as the meiofaunal samples. Grain-sizes of the upper 5 cm layer of sediments were measured by ANALYSETTE 22 MicroTec Plus laser diffraction (Ecological and geochemical center of the Geographical Faculty of Lomonosov Moscow State University). The silt–clay content (volume percent of particles < 63 μm) was calculated for particle size distribution^64^. Organic carbon was measured by dichromate oxidation (Ecological and Geochemical Center of the Geographical Faculty of Lomonosov Moscow State University).

Four cores per station from two separate sampler deployments were subsampled for meiobenthos using a 20 ml disposable syringe with a cut-off front end (inner diameter 2 cm). The subsamples were analyzed down to a depth of 5 cm. Subsamples for meiobenthos were fixed in a 4% formaldehyde filtered saltwater solution. In the laboratory, all meiobenthos subsamples were stained with Rose Bengal and washed through a sieve with a 40 μm mesh. The meiobenthos were extracted by centrifugation in Ludox^64^. All organisms retained on the 40 μm sieve were counted and sorted into major taxa. Per subsample, 100 nematodes (or all nematodes when the density < 100 individuals) were picked out at random and mounted on glycerin slides for identification to the genus level. The four feeding groups, distinguishing selective (1A) and non-selective (1B) deposit feeders, epistrate feeders (2A) and predators/omnivores (2B), based on buccal morphology, were used to investigate the trophic structure of the nematode assemblages^65^.

### Statistical analysis

We used the PAST software^66^ to describe differences in the spatial distribution of the nematode community between cores at each station and between the different stations. Similarities in nematodes composition (data for the uppermost 5 cm of sediments from all stations) were analyzed using cluster analysis. Correlation-based Principal Component Analysis (PCA) was used to reveal trends in environmental variables along the transect. Canonical correspondence analysis (CCA) was used to showing abundance distribution of meiobenthic taxa in relation to environmental variables^66^. Simper (Similarity Percentage) was used for assessing which genera were primarily responsible for an observed difference between groups of samples. The overall significance of the difference was assessed by analysis of similarities (ANOSIM). In the output table, nematode genera were sorted in descending order of their contribution to the group difference^66^. The number of genera (S), total number of individuals (*n*), dominance = 1 − Simpson index, Shannon index, and Pielou index were used as measures of genus diversity. Dominance is given on a range from 0 (all taxa are equally present) to 1 (one taxon dominates the community completely): D = ∑ (*n*_*i*_/*n*)^2^, where *n*_*i*_ is the number of individuals of taxon *i*. The Shannon index takes into account the number of individuals as well as the number of taxa and it varies from 0 for communities with only a single taxon to high values for communities with many taxa, each with few individuals: H =  − ∑ (n_i_/n) × ln (n_i_/n). The Pielou index measures the evenness with which individuals are divided among the taxa present^67^.

## References

[1] Stein R, Macdonald RW, Stein R, Macdonald RW (2004). Organic carbon budget: Arctic Ocean vs. global ocean. The Organic Carbon Cycle in the Arctic Ocean.

[2] Barber DG, Massom RA (2007). The role of sea ice in Arctic and Antarctic polynyas. Oceanogr. Ser..

[3] Sheremetevskiy AM (1987). Role of meiobenthos of the South Sakhalin shelf, Eastern Kamchatka, and Novosibirsk shallow water area. Issledovaniya Fauny Morei.

[4] Golikov AN (1990). Ecosystems of the New Siberian shoals and fauna of the Laptev Sea and adjacent waters of the Arctic Ocean (in Russian). Explor. Fauna Seas.

[5] Golikov AN (1994). Fauna of the East Siberian Sea. Part III. Explor. Fauna Seas.

[6] Sirenko BI, Denisenko SG (2010). Fauna of the East Siberian Sea, distribution patterns and structure of bottom communities. Explor. Fauna Seas.

[7] Sirenko BI (2001). List of species of free-living invertebrates of Eurasian Arctic seas and adjacent deep waters. Explor. Fauna Seas.

[8] Schmidt-Rhaesa A (2020). Handbook of Zoology: Gastrotricha, Cycloneuralia, Gnathifera.

[9] Udalov A (2021). Integrity of benthic assemblages along the arctic estuarine-coastal system. Ecol. Indic..

[10] Portnova D, Fedyaeva M, Udalov A, Tchesunov A (2019). Community structure of nematodes in the Laptev Sea shelf with notes on the lives of ice nematodes. Reg. Stud. Mar. Sci..

[11] Gallucci F, Moens T, Fonseca G (2009). Small-scale spatial patterns of meiobenthos in the Arctic deep sea. Mar. Biodivers..

[12] Lei Y, Stumm K, Volkenborn N, Wickham SA, Berninger UG (2010). Impact of Arenicola marina (Polychaeta) on the microbial assemblages and meiobenthos in a marine intertidal flat. Mar. Biol..

[13] Flint MV, Poyarkov SG, Rymsky-Korsakov NA (2018). Ecosystems of the Siberian Arctic Seas-2017 (Cruise 69 of the R/V Akademik Mstislav Keldysh). Oceanology.

[14] Garlitska LA, Azovsky AI (2016). Benthic harpacticoid copepods of the Yenisei Gulf and the adjacent shallow waters of the Kara Sea. J. Nat. Hist..

[15] Portnova D, Garlitska L, Udalov A, Kondar D (2017). Meiobenthos and nematode community in the Yenisei Bay and adjacent parts of the Kara Sea shelf. Oceanology.

[16] Carmack E, Polyakov I, Padman L, Fer I, Hunke E, Hutchings J, Melling H (2005). Toward quantifying the increasing role of oceanic heat in sea ice loss in the new Arctic. Bull. Am. Meteorol. Soc..

[17] Peterson BJ (2002). Increasing river discharge to the Arctic Ocean. Science.

[18] Polukhin A (2019). The role of river runoff in the Kara Sea surface layer acidification and carbonate system changes. ERL.

[19] Lisitzin AP (1994). Marginal filter of the oceans. Oceanology.

[20] Moens, T., Braeckman, U., Derycke, S., Fonseca, G., Gallucci, F., Gingold, R., Guilini, Katja, Ingles, J., Leduc, D., Vanaverbeke, J., Van Colen, C., Vanreusel, A, & Vincx, M. Ecology of free-living marine nematodes. In Volume 2 Nematoda, 109–152. De Gruyter (2013)

[21] Aller JY, Aller RC (1986). General characteristics of benthic faunas on the Amazon inner continental shelf with comparison to the shelf off the Changjiang River, East China Sea. Cont. Shelf Res..

[22] Soetaert K, Vincx M, Wittoeck J, Tulkens M (1995). Meiobenthic distribution and nematode community structure in five European estuaries. Hydrobiologia.

[23] Tank SE, Manizza M, Holmes RM (2012). The processing and impact of dissolved riverine nitrogen in the Arctic Ocean. Estuaries Coast.

[24] Galtsova VV, Lukina TG, Vladimirov MV (1994). Meiobenthos of Chaunskaya Bay, East Siberian Sea. Issledovaniya Fauny Morei.

[25] Coull BC (1999). Role of meiofauna in estuarine soft-bottom habitats*. Austral Ecol.

[26] Vincx, M., Meire, P., & Heip, C. The distribution of nematodes communities in the Southern Bight of the North Sea. *Cah Biol Mar*. **31**(1), 107–129 (1990).

[27] Vanaverbeke J, Gheskiere T, Steyaert M, Vincx M (2002). Nematode assemblages from subtidal sandbanks in the Southern Bight of the North Sea: effect of small sedimentological differences. J. Sea Res..

[28] Steyaert M (2003). The importance of fine-scale, vertical profiles in characterising nematode community structure. Estuar Coast Shelf Sci.

[29] Alves AS, Adão H, Patrício J, Neto JM, Costa MJ, Marques JC (2009). Spatial distribution of subtidal meiobenthos along estuarine gradients in two southern European estuaries (Portugal). J. Mar. Biol. Assoc. U. K..

[30] Garlitska LA, Chertoprud ES, Portnova DA, Azovsky AI (2019). Benthic harpacticoida of the Kara Sea: Species composition and bathymetrically related distribution. Oceanology.

[31] Huang D, Wang J, Zeng Q, Xiao J, Tian P, Fu S, Guo F, Niu W (2021). Preliminary study on community structures of meiofauna in the middle and eastern Chukchi Sea. Acta Oceanol. Sin..

[32] Giere O (2009). Meiobenthology: The Microscopic Motile Fauna in Aquatic Sediments.

[33] Semiletov I, Dudarev O, Luchin V, Charkin A, Shin K-H, Tanaka N (2005). The East Siberian Sea as a transition zone between Pacific-derived waters and Arctic shelf waters. Geophys. Res. Lett..

[34] Miroshnikov AY, Flint MV, Asadulina EE, Кravchishina MD, Luksha VL, Usacheva AA, Ryabchuk DV, Кomarov VB (2020). Ecological state and mineral-geochemical characteristics of the bottom sediments of the East Siberian Sea. Oceanology.

[35] Frontalini F (2018). The response of cultured meiofaunal and benthic foraminiferal communities to lead exposure: Results from mesocosm experiments. Environ. Toxicol. Chem..

[36] Fonseca G, Soltwedel T (2007). Deep-sea meiobenthic communities underneath the marginal ice zone off Eastern Greenland. Polar Biol..

[37] Portnova D, Polukhin A (2018). Meiobenthos of the eastern shelf of the Kara Sea compared with the meiobenthos of other parts of the sea. Reg. Stud. Mar. Sci..

[38] Alexeev DK, Galtsova VV (2012). Effect of radioactive pollution on the biodiversity of marine benthic ecosystems of the Russian Arctic shelf. Polar Sci..

[39] Grzelak K, Sørensen MV (2019). Diversity and community structure of kinorhynchs around Svalbard: first insights into spatial patterns and environmental drivers. Zool. Anz..

[40] Landers SC, Bassham RD, Miller JM, Ingels J, Nuria Sánchez N, Sørensen MV (2020). Kinorhynch communities from Alabama coastal waters. Mar. Biol. Res..

[41] Holovachov O (2007). New and known species of the genus Campylaimus Cobb, 1920 (Nematoda: Araeolaimida: Diplopeltidae) from North European marine habitats. Biodivers. Data J..

[42] Sharma J, Bluhm BA (2010). Diversity of larger free-living nematodes from macrobenthos (> 250 μm) in the Arctic deep-sea Canada Basin. Mar. Biodivers..

[43] Kotwicki L, Grzelak K, Bełdowski J (2016). Benthic communities in chemical munitions dumping site areas within the Baltic deeps with special focus on nematodes. Deep Sea Res. II.

[44] Netto SA, Pagliosa PR, Colling A, Fonseca AL, Brauk KM (2018). Benthic estuarine assemblages from the Southern Brazilian marine ecoregion. Braz. Estuaries..

[45] Broman, E., *et al.* Uncovering diversity and metabolic spectrum of animals in dead zone sediments. *Commun. Biol.* **3**(1), 1–12 (2020). 10.1038/s42003-020-0822-7 PMC7060179 32144383

[46] Zeppilli, D., *et al.* Characteristics of meiofauna in extreme marine ecosystems: a review. *Mar. Biodiver.* **48**(1), 35–71 (2018).

[47] Pérez-García JA (2018). Nematode diversity of freshwater and anchialine caves of Western Cuba. PBSW.

[48] Bezzubova EM, Seliverstova AM, Zamyatin IA, Romanova ND (2020). Heterotrophic bacterioplankton of the Laptev and East Siberian Sea shelf affected by freshwater inflow areas. Oceanology.

[49] Vanreusel A (2000). Meiobenthos of the central Arctic Ocean with special emphasis on the nematode community structure. Deep Sea Res. I.

[50] Tahseen Q (2012). Nematodes in aquatic environments: Adaptations and survival strategies. Biodivers. J..

[51] Williams WJ, Carmack EC (2015). The ‘interior’ shelves of the Arctic Ocean: Physical oceanographic setting, climatology and effects of sea-ice retreat on cross-shelf exchange. Prog. Ocean.

[52] Magritsky DV (2018). Long-term changes of river water inflow into the seas of the Russian Arctic sector. Polarforschung.

[53] Anderson LG (2011). East Siberian Sea, an Arctic region of very high biogeochemical activity. Biogeosciences.

[54] Dmitrienko IA (2010). Impact of the Arctic Ocean Atlantic water layer on Siberian shelf hydrography. J. Geophys. Res. Oceans..

[55] Stein R (2008). Arctic Ocean Sediments: Processes, PROXIES, and paleoenvironment.

[56] Petrova VI, Batova GI, Kursheva AV, Litvinenko IV (2010). Geochemistry of organic matter of bottom sediments in the rises of the central Arctic Ocean. Russ. Geol. Geophys..

[57] Millero FJ (1995). Thermodynamics of the carbon dioxide system in oceans. GCA.

[58] Pavlova GY (2008). Intercalibration of Bruevich’s method to determine the total alkalinity in seawater. Oceanology.

[59] Dickson, A. G. & Goyet, C. *Handbook of Methods for the Analysis of the Various Parameters of the Carbon Dioxide System in Sea Water. Version 2 (No. ORNL/CDIAC-74)* (1994).

[60] Dickson AG, Afghan JD, Anderson GC (2003). Reference materials for oceanic CO2 analysis: A method for the certification of total alkalinity. Mar. Chem..

[61] Lewis E, Wallace DWR (1998). Program Developed for CO2 System Calculations. ORNL/CDIAC-105.

[62] Shiklomanov, A. I., Holmes, J. W., McClelland, S. E., Tank, R. & Spencer, G.M. *Arctic Great Rivers Observatory. Discharge Dataset, Version 20200801* (2020).

[63] Niemistö L (1974). A gravity corer for studies of soft sediments. Merentutkimuslait. Julk./Havsforskningsinst. Skr..

[64] Eleftheriou A (2013). Methods for the Study of Marine Benthos.

[65] Wieser W (1953). Beziehungen zwischen Mundhöhlengestalt, Ernährungsweise und Vorkommen bei freilebenden, marinen Nematoden. Ark. Zool..

[66] Hammer Ø, Harper DAT, Ryan PD (2001). PAST paleontological statistics software package for education and data analysis. Palaeontol. Electron..

[67] Heip C, Herman P (2001). Indices of diversity and evenness. Oceanis.

